# Extraction of Phenolic-Rich Fractions from *Borago officinalis* By-Products with Antioxidant and Antimicrobial Activities

**DOI:** 10.3390/foods15111917

**Published:** 2026-05-28

**Authors:** Eva Tejedor-Calvo, Adrián Orihuela-Jaro, Laura Jaime, Laura de la Fuente-Nieto, Diego Morales

**Affiliations:** 1 Plant Science Department, Agrifood Research and Technology Centre of Aragon (CITA), Avda. Montañana 930, 50059 Zaragoza, Spain; etejedor@unizar.es; 2 Departmental Section of Galenic Pharmacy and Food Technology, Veterinary Faculty, Complutense University of Madrid, Av. Puerta del Hierro, s/n, 28040 Madrid, Spain; adriorih@ucm.es; 3 Department of Production and Characterization of Novel Foods, Food Science Research Institute (CIAL) (UAM + CSIC), Universidad Autónoma de Madrid, C/Nicolas Cabrera 9, 28049 Madrid, Spain; laura.jaime@uam.es (L.J.); laura.fuente@estudiante.uam.es (L.d.l.F.-N.); 4 Departmental Section of Food Science, Department of Applied Physical Chemistry, Faculty of Science, Universidad Autónoma de Madrid, Ave. Francisco Tomás y Valiente, n/a, 28049 Madrid, Spain

**Keywords:** borage, borage by-products, phenolic compounds, ultrasound-assisted extraction, antioxidant activity, antimicrobial activity

## Abstract

Borage (*Borago officinalis*) is an herbaceous plant recognized for its bioactive properties and, particularly, for its culinary use in Mediterranean countries. In gastronomy, the petioles are generally consumed, while a substantial proportion of leaves and other tissues are discarded. These borage by-products (BBPs) constitute a valuable source of phenolic compounds with potential biological activities, including antioxidant and antimicrobial effects. Accordingly, this study evaluated both conventional solid–liquid extractions and an advanced technology, ultrasound-assisted extraction (UAE), to obtain bioactive BBP fractions. Different hydroethanolic mixtures were initially assessed. Although extractions using 25% ethanol did not yield the highest total phenolic content (TPC), they produced extracts with the strongest antioxidant capacity, as reflected by the highest Trolox equivalent antioxidant capacity (TEAC) values against DPPH^●^ and ABTS^●+^ (24 and 117 µmol/g). Response surface methodology (RSM) was employed to establish the most suitable extraction time and temperature (78 min, 70 °C) to maximize extraction yield, TPC, and radical-scavenging activity. In contrast, UAE enabled the use of milder conditions (45 min, 25 °C) while still achieving comparable TPC and TEAC values (15%, 29 and 246 µmol/g). Phenolic characterization of selected extracts revealed the presence of nine compounds, with epigallocatechin and rosmarinic acid identified as the major constituents. These extracts exhibited antibacterial activity against *Staphylococcus aureus* and *Escherichia coli*, whereas no inhibitory effect was observed against *Listeria innocua*. Overall, these results highlight the bioactive potential of BBP extracts and encourage further investigations into their functional properties, as well as sensory and consumer acceptance studies.

## 1. Introduction

Borage (*Borago officinalis* L.) is an annual herbaceous plant belonging to the family Boraginaceae that has been traditionally cultivated for culinary and medicinal uses. Although the species is distributed throughout much of the Mediterranean basin and temperate Europe, its gastronomic utilization is particularly notable in Germany, France, Italy and Greece, and holds a distinctive relevance in Spanish food culture. Within Spain, its consumption is especially concentrated in Aragón, La Rioja, Navarra and the Ebro Valley, where it is commonly prepared by boiling or steaming and incorporated into a variety of meals, ranging from simple preparations dressed with olive oil or sauces to more elaborate soups, stews, omelettes, vegetable-based fillings, etc. [1,2].

Currently, borage is commercially produced as an oilseed crop, and the edible parts of the plant that are utilized in the previously mentioned dishes consist mainly of the basal leaf petioles that are harvested in fully developed plants before flowering. As a consequence of this selective processing, most of the leaf biomass, including the leaf blades, is usually discarded; therefore, approximately 60% of the total plant material is considered a by-product. This proportion may vary depending on the specific conditions of cultivation and developmental stage [1,3]. Several factors account for the selection of only this portion of the plant, most notably the presence of trichomes, which significantly impair the sensory attributes of borage, especially texture. In addition, some reports have indicated significant content of toxic pyrrolizidine alkaloids (PAs) in green leaves, further supporting the exclusion of these tissues [4]. In any case, it would be advisable for products intended for human consumption that include borage to be analyzed for their PA content, in order to ensure that they remain below the reference value of 237 µg/kg body weight per day established by the EFSA CONTAM Panel [5,6].

Given the impact of this by-product generation, mitigation strategies based on their valorisation are warranted. In this context, the richness of borage leaves in bioactive compounds with potentially beneficial effects on human health is well recognized. Beyond fatty acids and vitamins, these tissues are particularly notable for their phenolic profile, which includes flavonoids such as astragalin and phenolic acids (caffeic, syringic, and rosmarinic acids, among others) [7,8]. These phenolic species have exhibited a range of relevant activities, including antioxidant, antimicrobial, hypoglycemic, hypolipidemic, anti-inflammatory, and antiproliferative effects [9,10]. However, although preclinical research on borage (and derived fractions or products) is growing, it has not yet been incorporated into clinical practice. Many bioactivities have been tested in vitro and in animal models, but human studies have been limited to specific conditions (atopic dermatitis, premenstrual syndrome, metabolic disorders, etc.). Therefore, it still requires proper standardization and deeper clinical validation [11].

Moreover, for phenolic compounds to exert their biological actions, they must be present at sufficient concentrations; to this end, different extraction technologies may be employed, ranging from conventional approaches, such as solid–liquid extraction using different solvents (most commonly water, ethanol, or hydroalcoholic mixtures) at varying temperatures, to more advanced technologies involving the application of pulsed electric fields, supercritical fluids, or ultrasonic waves [2]. In the latter case, it is well established that ultrasound-assisted extraction (UAE) enables the implosion of cavitation-generated bubbles, leading to the disruption of cell membranes and cell walls, thereby enhancing extraction yields and the recovery of intracellular compounds. This mechanism is particularly suitable for applications involving plant cells, such as those present in borage [12,13].

Taking all these considerations into account, this work targeted borage tissues that are usually discarded by food companies and not applied to any alternative use, with the aim of evaluating different extraction methodologies for obtaining fractions derived from these borage by-products (BBPs) with a high content of phenolic compounds and in vitro antioxidant and antimicrobial activities. For this purpose, conventional extractions using hydroethanolic mixtures were investigated, selecting the most appropriate ethanol percentage as well as the optimal extraction temperature and time by means of Response Surface Methodology (RSM). In addition, the potential to reduce extraction temperature and time through the application of ultrasound was also examined. The novelty and relevance of this study lie not only in the optimization of environmentally friendly extraction methods, but also in the valorization of BBPs that are massively wasted in agri-food systems, through a deeper assessment of their content of bioactive compounds and their potential biological activities, thus laying the groundwork for future studies to validate these properties, which may prove key in the design of functional foods or nutraceuticals based on fractions derived from BBPs, an area of research that, to date, remains underexplored.

For this purpose, this work used BBPs that have not been the subject of previous studies as raw materials, specifically leaves and parts of the stems that are separated in greengroceries and are not marketed, whereas previously published studies have mainly focused on other plant tissues and parts (leaves, flowers, seeds, roots) or on other by-products (seedcakes) of a different nature from those evaluated here [2]. Additionally, UAE procedures have been evaluated in this research with the aim of applying them to a plant species in which this technique is clearly underutilized, as there are only two studies focused on obtaining borage fractions using UAE, both of which used the flowers as raw material [14,15]. The extracted fractions were subjected to phenolic characterization, yielding profiles that differed from those reported for other borage-derived samples recovered from different sources or obtained through other technologies [16,17]. Finally, although biological activities such as antioxidant and antimicrobial effects have been analysed in some fractions obtained from *B. officinalis* [18,19,20,21], an initial screening of the antioxidant and antimicrobial potential of the extracts collected in the present study has been carried out. To the best of our knowledge, this is the first report of biological activity results obtained using this type of combination of BBP and extraction technique; however, as these are preliminary in vitro assays, the results should be interpreted with caution. Further validation in cellular and in vivo models is required to advance this line of research and to reach more robust and meaningful conclusions.

## 2. Materials and Methods

### 2.1. Biological Material

Borage by-products (BBPs), specifically leaves and parts of the stems, were obtained from a local greengrocery (Zaragoza, Spain), where they are routinely discarded. These BBPs were previously lyophilized, ground, and sieved to achieve a particle size below 0.5 mm.

For the antimicrobial activity studies, indicator strains were used, including non-pathogenic laboratory strains and reference strains from species associated with human pathogenicity: *Escherichia coli* (DH5α strain) (Thermo Fisher Scientific, Waltham, MA, USA), *Staphylococcus aureus* (CECT 86–NCTC 8532 strain), and *Listeria innocua* (CECT 8848–SA1 strain) from the Spanish Type Culture Collection (CECT) were employed.

### 2.2. Reagents

Solvents (HPLC grade), such as methanol and absolute ethanol, were acquired from Panreac (Barcelona, Spain), as well as sodium carbonate (Na_2_CO_3_). Hydrochloric acid (HCl, 37%), Folin–Ciocalteu’s phenol reagent, 2,2′-azino-bis(3-ethlybenzothiazoline-6-sulphonic acid (ABTS), potassium persulfate, gallic acid, 2,2-diphenyl-1-picrylhydrazyl (DPPH), and Trolox were purchased from Sigma-Aldrich Quimica (Madrid, Spain). Brain Heart Infusion (BHI) broth and bacteriological agar were obtained from Condalab (Torrejón de Ardoz, Spain). Acetonitrile (HPLC grade), dimethyl sulfoxide (DMSO) (HPLC grade) and formic acid (purity ≥ 99%) were from Macron Chemicals Fine (Gliwice, Poland). Chromatographic reference standards (purity ≥ 95%) such as epigallocatechin, caffeic acid and astragalin were purchased at Extrasynthese S.A. (Genay, France), gallic acid was acquired at Sigma-Aldrich Quimica (Madrid, Spain), whereas rosmarinic acid, lithospermic acid, and salvianolic acid were from Phytolab (Madrid, Spain).

### 2.3. Conventional Extractions of BBP Fractions Using Ethanol-Water Mixtures

Based on laboratory tests to evaluate the feasibility of extracting borage fractions and on published data regarding solute-to-solvent ratios [2], BBP powder (<0.5 mm) was mixed (25 g/L) with aqueous ethanol solutions at different concentrations (0, 25, 50, 75, and 100% *v*/*v*). The mixtures were subjected to vigorous magnetic stirring for 30 min at room temperature (25 °C). Subsequently, the samples were centrifuged at 10,000 rpm for 2 min, and the supernatants were collected for the analyses described below. Until use, the supernatants were stored at −20 °C. All extraction procedures were carried out in duplicate.

### 2.4. Response Surface Methodology (RSM) Experimental Design for the Extraction of BBP Fractions

For this research, a full factorial three-level experimental design (3^2^) was selected once the most suitable solvent was chosen (25% ethanol). Two factors were analyzed: time (5–120 min) and temperature (25–70 °C). The response variables investigated were extraction yield (dry weight), total phenolic content of the extracts, and their radical (DPPH^●^ and ABTS^+●^) scavenging activity, calculated as TEAC values. In total, eleven experiments were conducted in randomized order: nine points of the factorial design and two additional center points to account for experimental error. The experimental matrix design is detailed in Table 1. Optimal extraction conditions were estimated by multiple linear regressions using Statgraphics Centurion 19 software (Statpoint Technologies, Warrenton, VA, USA). Each response was transformed into an individual desirability function, and the overall optimized desirability was calculated as the geometric mean of all individual desirabilities, considering all the investigated responses and exclusively the TEAC values from in vitro antioxidant assays. Validation experiments were performed at the predicted optimum conditions.

### 2.5. Ultrasound-Assisted Extraction (UAE) of BBP Fractions

Similarly, and in addition to conventional extractions, UAEs were also performed. The solute-solvent ratio was the same (25 g/L), and the extraction times applied were 5, 15, 30, and 45 min. The extractions were performed (in duplicate) using an UP400S (400 W, 24 kHz) ultrasonic processor (Hielscher Ultrasonics, Teltow, Germany) operating at 60% amplitude, following the method described by de las Nieves Siles-Sánchez et al. (2025) [12]. The whole process was carried out at 25 °C, using an ice-cooled water bath when necessary to control temperature increases. Subsequently, the samples were centrifuged at 10,000 rpm for 2 min, and the supernatants were stored at −20 °C until use.

### 2.6. Determination of Total Phenolic Content (TPC)

The TPC of the extracted fractions was quantified using the Folin–Ciocalteu method, following the protocol described in Tejedor-Calvo and Morales (2023) [22]. Briefly, the samples (50 µL) were mixed with 1.48% HCl (133 µL) and methanol (67 µL) and centrifuged (2 min, 10,000 *g* rpm). Aliquots of the supernatants (50 µL) were added to Na_2_CO_3_ (2% *w*/*v*, 1 mL), subjected to vigorous magnetic stirring, incubated at room temperature for 3 min, and then mixed with Folin–Ciocalteu reagent (25 µL). After incubation for 30 min, absorbance was measured at 750 nm in a Genesys 10 UV spectrophotometer (Thermo Fisher Scientific, Madrid, Spain). Gallic acid (0.015 to 0.5 mg/mL) was used as the standard for quantification.

### 2.7. HPLC-DAD Phenolic Compounds Analysis

Following the method reported by Fernández-Jalao et al. (2025) [23], the phenolic compounds present in the samples were characterized using an HPLC-DAD 1260 Infinity series system (Agilent Technologies Inc., Santa Clara, CA, USA). The analysis was performed using an ACE Excel 3 Super C18 column (150 mm × 4.6 mm, 3 μm particle size) (VWR International, Llinars del Vallés, Spain), coupled to a guard column (ACE Excel 3 C18, 10 mm × 3 mm) (Symta, Madrid, España), with the column temperature maintained at 35 °C. A binary mobile phase was employed, consisting of milli-Q water acidified with 0.1% (*v*/*v*) formic acid as solvent A and 100% acetonitrile with 0.1% (*v*/*v*) formic acid as solvent B, operating at a constant flow rate of 0.5 mL/min. Prior to injection (20 μL), the samples were dissolved in DMSO and filtered through 0.45 μm PVDF filters (Symta, Spain). Finally, the quantification of phenolic compounds was carried out using calibration curves constructed with commercial standards (HPLC purity ≥ 95%). The following regression equations were obtained and applied using standard solutions prepared at concentrations ranging from 0.0001 to 1 mg/mL: gallic acid y = 105,746x − 6.0637 (R^2^ = 0.9998); epigallocatechin y = 5908x − 113.326 (R^2^ = 0.9998); caffeic acid y = 226,201x − 25.963 (R^2^ = 0.9995); astragalin y = 60,984x + 77.419 (R^2^ = 0.999); rosmarinic acid y = 107,968x − 146.66 (R^2^ = 0.999); lithospermic acid y = 59,823x − 17.399 (R^2^ = 0.9998); and salvianolic acid y = 32,861x − 9.6419 (R^2^ = 1). Gallic acid and astragalin were used as reference compounds for benzoic acid derivatives and flavonols, respectively.

### 2.8. DPPH^●^ and ABTS^●+^ Scavenging Activities

The obtained BPP extracts were diluted (6.25–100 µL/mL) and assayed (in duplicate) for their DPPH^●^ and ABTS^●+^ scavenging activities. The dilutions were mixed with a methanolic DPPH^●^ solution (76 µM) or an aqueous ABTS^●+^ solution (7 mM; the ABTS radical was chemically generated using 2.47 mM potassium persulfate), according to Mau et al. (2001) and Re et al. (1999), respectively, as adapted by Morales et al. (2018) [24,25,26]. In the case of the DPPH^●^ assay, absorbance at 517 nm was read after 30 min incubation at room temperature in darkness. For the ABTS^●+^ assay, absorbance was measured at 734 nm after 15 min of incubation under the same conditions. For both radicals, the half maximal inhibitory concentration (IC_50_) values were determined from the linear correlation obtained using increasing sample concentrations. Linear regression equations were fitted according to the model “percentage of scavenged radical = a *×* extract concentration + b”, where ‘a’ is the slope of the line and ‘b’ is the y-intercept. The IC_50_ values of the extracts and Trolox were compared to express the results as their TEAC (Trolox equivalent antioxidant capacity) values.

### 2.9. Antimicrobial Activity

Antimicrobial activity of selected BBP extracts was determined using the disc diffusion method. Each microorganism (*S. aureus*, *E. coli* and *L. innocua*; 10^5^ ufc/mL) was resuspended in a semi-solid BHI medium containing bacteriological agar (0.75% *w*/*v*) and poured into sterile Petri dishes. Sterile Oxoid^TM^ blank test discs (Thermo Fischer Scientific, Madrid, Spain) were placed on the surface of the inoculated agar, and each disc was impregnated with the corresponding extract (0.1 mg) dissolved in 25% ethanol. Gentamicin (10 µg) was used as positive antimicrobial control (Oxoid^TM^ gentamicin test discs, Thermo Fischer Scientific, Madrid, Spain) and 25% ethanol (20 µL) as negative control. The plates were then incubated for 48 h at 37 °C. Each sample was tested in triplicate, and antimicrobial activity was evaluated by measuring the diameter (mm) of the inhibition zones.

### 2.10. Statistical Analysis

Differences were evaluated at a 95% confidence level (*p* ≤ 0.05) using a one-way analysis of variance (ANOVA) followed by the Tukey multiple comparison test. Statistical analysis was performed using GraphPad Prism version 9.5.1 (GraphPad Software, San Diego, CA, USA).

## 3. Results and Discussion

### 3.1. Effect of the Solvent: Extraction Yield, Total Phenolic Content and In Vitro Antioxidant Activity

**Figure 1:**
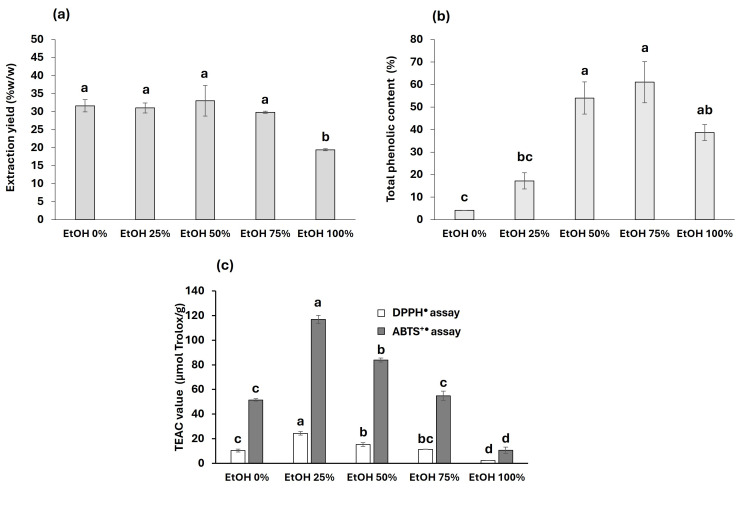
Effect of the utilized solvent on (**a**) extraction yield (% *w*/*w*, g extract per 100 g BBP), (**b**) total phenolic content (%, g total phenolic compounds per 100 g extract) and (**c**) DPPH^●^ and ABTS^+●^ scavenging capacity, expressed as TEAC values (µmol Trolox/g extract). Different letters (a–d) indicate statistical significance (*p* ≤ 0.05) among solvents on each analyzed parameter.

Due to the diversity of phenolic compounds with different polarities present in borage and BBPs, hydroalcoholic mixtures containing varying percentages of ethanol or methanol are commonly used to achieve their extraction [7,27]. In the present study, water and absolute ethanol (100%) were used, as well as different hydroethanolic mixtures (25, 50, and 75% ethanol). This initial screening was required because the phenolic characterization of these specific BBPs, composed of leaves and portions of the stems, had not been reported prior to the present study. In terms of extraction yield (g extract/100 g BBP dry weight), the use of absolute ethanol resulted in the lowest value (19%), whereas no significant differences were observed among the other solvents, with yields ranging from 30 to 33%. These results are comparable to those reported by Zemmouri et al. (2019) using borage leaves, achieving extraction yields of up to 45% using boiling water, which decreased to 5% when the ethanol concentration was increased to 80% [18]. This suggests the presence of high levels of polar and intermediate-polarity compounds in borage and BBPs, which require the use of water in the extracting solvent. The higher yield shown by Zemmouri et al. (2019) for aqueous extraction [18] may be attributed to the higher temperature employed, as the temperature used for the extractions in this part of the present research was 25 °C (Figure 1a).

In contrast, the total phenolic content (TPC) of the extracts varied more markedly depending on the solvent used (Figure 1b). Extracts obtained utilizing water exhibited a TPC of only 4%, which was significantly lower than those achieved with hydroethanolic mixtures, whose values ranged from 17 to 61%. The highest TPC values were observed for extractions performed with 75% ethanol (61%) and 50% ethanol (54%). These differences are consistent with those described in previous studies: Segovia et al. (2014) reported a higher TPC in extracts from borage leaves applying maceration with 52% ethanol (3%) compared to boiling water (2%), while Zemmouri et al. (2019) obtained TPC values of 9% using 80% ethanol and only 3.5% with boiling water [18,27]. In both cases, the TPC levels reported were significantly lower than those obtained in the present study, which may be attributed to differences in the initial TPC of the raw material, directly influenced by environmental conditions, cultivation practices, processing, and storage of borage [28].

Although the extracts obtained with 25% ethanol did not exhibit the highest TPC, they showed the greatest scavenging capacity against both DPPH^●^ and ABTS^●+^ radicals (Figure 1c). Specifically, the TEAC values were 24 and 117 µmol Trolox equivalents/g for DPPH^●^ and ABTS^●+^, respectively. This behaviour may be attributed to the presence of specific phenolic compounds with higher antioxidant activity, as will be discussed later, or other non-phenolic antioxidants; however, these single compound–activity relationships must be validated in further studies with isolated molecules. Related to this, weak correlations were found between TPC and the TEAC values obtained from the DPPH^●^ and ABTS^●+^ assays when considering all extracts analyzed in this study (R^2^ = 0.22 and R^2^ = 0.21, respectively). In all cases, TEAC values were higher in the ABTS^●+^ assay than in the DPPH^●^ assay, which may be attributed to the predominance of more hydrophilic antioxidant molecules in BBPs that interact more efficiently with ABTS^●+^ radical in an aqueous environment than with DPPH^●^ in a methanolic medium [26]. Indeed, a consistent correlation (R^2^ = 0.99 within these samples; R^2^ = 0.82 within all the extracts in this study) was observed in which TEAC values obtained with the ABTS^●+^ assay were between 4.6- and 5.6-fold higher than those obtained with the DPPH^●^ assay.

When these results are compared with those reported for other borage and BBP extracts, it can be noted that Zemmouri et al. (2019) obtained fractions with higher DPPH^●^ scavenging capacity by extracting borage leaves with 80% ethanol, achieving an IC_50_ = 0.09 mg/mL, which is significantly lower than that calculated for the 25% ethanol extract in the present study (0.72 mg/mL). This difference may be explained by the higher ethanol concentration and the longer extraction time (24 h) used by these authors [18] but also by the use of leaves instead of BBPs (leaves together with portions of stems), which may lead to differences in in vitro antioxidant potential. DPPH^●^ scavenging capacity depends not only on the extraction method but also on the borage raw material: IC_50_ values of 0.21 and 0.65 mg/mL were reported for extracts obtained with 50% ethanol from borage seed cakes (a by-product of oil processing) and from borage flowers using absolute ethanol, respectively [19,29]. In the case of the extracts derived from seed cakes, ABTS^●+^ scavenging activity corresponded to an IC_50_ = 0.18 mg/mL, which is slightly lower than that obtained for the BBP extract prepared with 25% ethanol (IC_50_ = 0.12 mg/mL), although these by-products were derived from significantly different tissues and plant parts [19].

Taking into account the results obtained and prioritizing the bioactive -specifically antioxidant- potential of the extracts, 25% ethanol was established as the most suitable solvent and was therefore used for subsequent analyses.

Considering the nature of this solvent and the polarity of PAs, it is important to note that although the extraction of these molecules typically requires low pH and the presence of acids, the co-extraction of these potentially toxic compounds is still possible when using this type of hydroalcoholic mixture. In fact, although not specifically for these BBPs, the extraction of PAs using methanol-water mixtures has been reported in *B. officinalis* seed oil, and ethanol-water mixtures have also been used for PA extraction in other Boraginaceae species [30,31,32]. The extracted amounts are usually low and often require additional concentration or clean-up steps prior to analysis. However, the lack of quantitative data for BBPs and the clinical relevance of the compounds make it essential to quantify PA levels in any extracts or fractions intended for potential incorporation into food products. Such quantification would allow determining whether the intake of a food or nutraceutical containing the extract remains below the limit mentioned in Section 1 of 237 µg of PAs per kg of body weight per day [5,6]. The potential adverse health effects associated with these alkaloids include hepatotoxic, mutagenic, and carcinogenic actions, representing a significant health risk [31]. Although the present study, being preliminary in nature, did not focus on quantifying PAs in all obtained extracts, as previously noted, these analyses must be carried out in future work for any extracts selected for further investigation.

### 3.2. RSM Analysis of Extraction Time and Temperature

A 3^2^ factorial design was applied, setting a temperature range from room temperature (25 °C) to a significantly higher temperature (70 °C) that did not pose operational difficulties when working with hydroethanolic solvents, as well as an extraction time range from short periods (5 min) to longer periods (120 min). These times were considered sufficient for the extraction of phenolic compounds and antioxidants from borage, based on previous experimental results and published literature, while not being excessively long to ensure time- and cost-efficient processing [2].

Considering the results obtained under the different experimental conditions, it can be observed that the lowest extraction yields were recorded at 25 °C, with values of 13.2 and 16.8% for extraction times of 5 and 62.5 min, respectively (Table 1). In contrast, the highest yields corresponded to the central point of the factorial design, which was performed in triplicate (62.5 min and 47.5 °C), ranging from 25.8 to 26.8%. This may suggest that intermediate values of time and temperature favour the extraction of a greater amount of material, whereas longer extraction times and higher temperatures could promote denaturation, aggregation, and precipitation phenomena [33,34]. Regarding TPC, the highest value was recorded for the extract obtained at 25 °C for 62.5 min, suggesting that higher extraction temperatures did not necessarily lead to a higher concentration of phenolic compounds in the extract. This may be due either to degradation or aggregation phenomena or to the co-extraction of non-phenolic compounds that reduce the relative proportion of (poly)phenols in the extracted fractions [33,35]. With respect to TEAC values, the same trend was maintained, with extracts exhibiting a greater scavenging capacity against ABTS^●+^ than against DPPH^●^ (4.3–5.3-fold higher in this case). The highest TEAC values against both free radicals were observed for the experiment conducted at 70 °C for 62.5 min. These experimental conditions appeared to be the most suitable among those tested for the extraction of free radical–scavenger phenolic compounds, as well as, likely, other non-phenolic antioxidant molecules present in borage, as described in the literature (e.g., fatty acids, carotenoids, tocopherols, ascorbic acid, etc.), although specific analysis must be carried out to confirm their presence and concentrations [29,36].

Overall, RSM analysis indicated that the optimal conditions to maximize the investigated responses (extraction yield, TPC, and TEAC values) were an extraction time of 78 min and a temperature of 70 °C. When all responses were simultaneously maximized, the overall desirability reached 0.63, indicating a moderate and limited level of optimization. In contrast, the desirability increased to 0.89 when only the TEAC values were considered, reflecting substantially improved optimization under this more specific criterion and the high impact on TEAC values-related responses. Moreover, the calculated R^2^ for ABTS^●+^ and DPPH^●^ TEAC values analysis was 0.81 and 0.78, respectively, while for the extraction yield and TPC was 0.74 and 0.43, respectively. The high R^2^ obtained for the TEAC values suggested that the fitted model explained a considerable proportion of the observed variability. However, the adjusted R^2^ decreased after correction for the degrees of freedom (0.62 and 0.56 for ABTS^●+^ and DPPH^●^ TEAC values, respectively), indicating that part of the apparent explanatory power may be attributable to model complexity rather than true predictive relevance. More importantly, the predicted R^2^ was 0, accompanied by a substantially elevated Predicted Residual Sum of Squares (PRESS) value (Supplementary Tables S4 and S5). This combination indicates that, despite the satisfactory fit to the experimental data, the model exhibited essentially no predictive capability for new observations. The regression equations fitting to the data were (*t* = extraction time; *T =* extraction temperature):

Extraction yield = −5.21505 + 0.052873 *t* + 1.04264 *T* − 0.000140086 *t*^2^ − 0.000231884 *t T* − 0.00960624 *T*^2^

TPC = 38.3546 − 0.0158241 *t* − 0.914294 *T* − 0.000486161 *t*^2^ + 0.00124638 *t T* + 0.00757063 *T*^2^

DPPH^●^ TEAC value = 67.1222 − 0.00121998 *t* − 1.27601 *T* − 0.00212102 *t*^2^ + 0.00457971 *t T* + 0.0113528 *T*^2^

ABTS^● +^TEAC value = 333.41 + 0.155601 *t* − 6.19146 *T* − 0.0131871 *t*^2^ + 0.028143 *t T* + 0.0493926 *T*^2^

According to the analysis of the variance (ANOVA) results, the significant factors and interactions identified (*p* < 0.05) were the quadratic temperature term (*T*^2^) for extraction yield (*p* = 0.046), the interaction between time and temperature (*t T*) for ABTS^● +^ TEAC value (*p* = 0.024) and the quadratic time term (*t*^2^) for ABTS^● +^TEAC value (*p* = 0.028).

**Figure 2:**
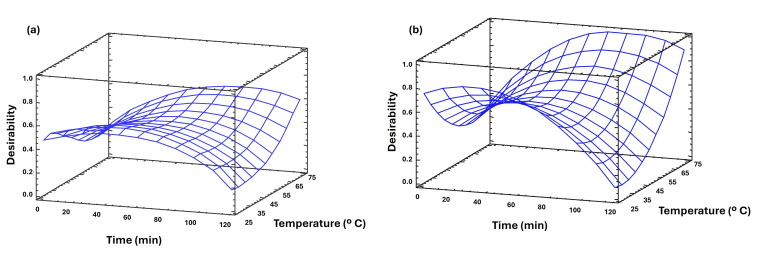
Response surface 3D plots (desirability function) for BBP extractions for (**a**) all responses investigated (extraction yield -% *w*/*w*-, total phenolic content -%-, DPPH^●^ and ABTS^+●^ TEAC values -µmol/g-) and (**b**) specifically radical scavenging activities (DPPH^●^ and ABTS^+●^ TEAC values -µmol/g-).

These results highlight the strong influence of antioxidant capacity among the investigated responses, which explains the clear and direct effect of temperature (70 °C as optimal value), but also of extraction times. However, these times should not be excessively long (78 min) to prevent a decrease in antioxidant values due to the aforementioned phenomena, such as degradation, aggregation, and precipitation events (Figure 2).

From a statistical perspective, these results suggest that the model may be overfitted and should therefore be interpreted with caution. While it may be suitable for describing trends within the experimental dataset, its reliability as a predictive or optimization tool is limited. Consequently, the identified optimal conditions should be considered provisional and require external experimental validation before being regarded as robust or generalizable (Supplementary Tables S1–S5).

### 3.3. Ultrasound-Assisted Extraction (UAE) to Obtain Antioxidant BBP Fractions

**Figure 3:**
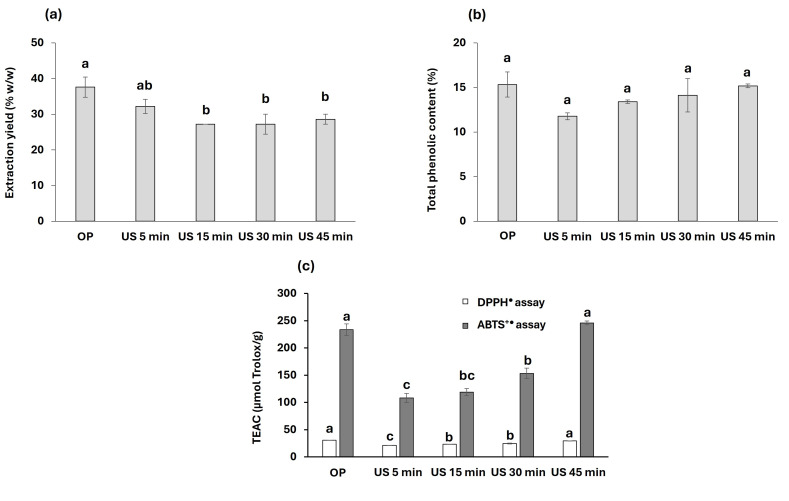
Effect of ultrasound-assisted extraction applied at different times (5–45 min) on (**a**) extraction yield (% *w*/*w*, g extract per 100 g BBP), (**b**) total phenolic content (%, g total phenolic compounds per 100 g extract) and (**c**) DPPH^●^ and ABTS^+●^ scavenging capacity, expressed as TEAC values (µmol Trolox/g extract) and comparison with the results observed for the extraction at the optimal conditions calculated through the Response Surface Methodology approach, i.e., 70 °C and 78 min (OP). Different letters (a–c) indicate statistical significance (*p* ≤ 0.05) on each analyzed parameter.

Once an extraction time of 78 min and a temperature of 70 °C were established as the most suitable conditions to obtain antioxidant fractions from BBPs by means of conventional solid–liquid extraction (as well as 25% ethanol as solvent), the objective was to assess the feasibility of applying UAE as an advanced technology capable of operating at shorter processing times and lower temperatures, thereby enabling the development of a more cost- and time-efficient process. Before analyzing the characteristics of UAE fractions, validation extraction experiments were performed under the optimal conditions (OP: 78 min, 70 °C). The results obtained were close to those predicted by the model for TPC and ABTS^●+^ TEAC values (15.33% vs. 14.06% and 233.69 µg/mol vs. 227.65 µg/mol, respectively) but differed significantly for extraction yield and DPPH^●^ TEAC values (37.6% vs. 22.7% and 30.71 µg/mol vs. 45.44 µg/mol, respectively) (Table 1, Figure 3). These differences may be attributed to the limited predictive capacity of the model, as discussed in the previous section.

When the results obtained using ultrasound at room temperature over a time range of 5 to 45 min were evaluated (Figure 3), it was observed that although the extraction yield (g extract/100 g BBP) decreased (27–32%) relative to that achieved at 70 °C and 78 min (38%) without ultrasound, no significant reduction in the TPC of the resulting extracts was detected. Notably, the extract produced by UAE after 45 min exhibited TEAC values comparable to those obtained by conventional extraction under the optimal design conditions, both against DPPH^●^ (29 and 31 µmol/g) and ABTS^●+^ (246 and 234 µmol/g). These findings confirm that UAE allows a reduction in operating temperature (from 70 °C to 25 °C) and extraction time (from 78 min to 45 min), corresponding to decreases of approximately 64% and 42%, respectively. Such reductions are expected to significantly lower energy consumption by avoiding heating requirements and shortening processing time, leading to a more streamlined and optimized procedure. Consequently, UAE is regarded as an environmentally friendly technology and, moreover, these specific protocols allow operation under mild thermal conditions that do not compromise the stability and bioactivity of the target compounds, in this case (poly)phenols and other antioxidant molecules present in BBPs. In addition, UAE is compatible with food-grade solvents and sustainable processing strategies, minimizes waste generation, and can be readily integrated into industrial systems, thus representing an appropriate approach aligned with the principles of green chemistry for the extraction of plant-derived compounds such as the aforementioned (poly)phenols from borage [2,8,33]. Indeed, previous studies have already reported the application of UAE to *Borago officinalis* flowers to obtain fractions enriched not only in (poly)phenols but also in other bioactive constituents, including ascorbic acid and certain fatty acids [14,15]. Nevertheless, aside from these two studies focusing on flowers, it is noteworthy that this technology remains largely underutilized when applied to other borage tissues, such as leaves or additional by-products. To the best of our knowledge, the current study is the first one in which UAE is used to extract bioactive compounds from BBPs, including leaves and stems.

### 3.4. Identification and Quantification of Specific Phenolic Compounds in BPP Extracts

To investigate specific differences in the phenolic profile of the extracts obtained, selected samples were analyzed, namely those produced using 50% and 75% ethanol, which yielded the highest TPC values according to the Folin–Ciocalteu method. In addition, the extract obtained under the optimal conditions of the factorial design (70 °C, 78 min), aimed at maximizing TPC and radical scavenging capacity, was evaluated together with two UAE-derived extracts corresponding to the shortest (5 min) and longest (45 min) extraction times (Table 2).

Thus, based on the sum of phenolic compounds detected and quantified by HPLC-DAD, it was confirmed that extracts obtained with higher ethanol proportions (50% and 75%) exhibited a greater content of phenolics than those extracted with 25% ethanol. These results were consistent with those reported using the Folin–Ciocalteu assay (R^2^ = 0.95). Nevertheless, the colorimetric method yielded higher values overall, which may be attributed to overestimations inherent to the Folin–Ciocalteu protocol, as the reagent can also react with other molecules such as sugars or amino acids, among others. Additionally, to a lesser extent, these discrepancies may be due to the inability to mathematically account for phenolic species present below the limits of detection and/or quantification [12].

A total of 9 compounds were detected and quantified, with epigallocatechin (Rt = 13.462 min) and rosmarinic acid (Rt = 29.204 min) being the predominant constituents. Other identified compounds by comparison with authentic standards included caffeic acid (Rt = 18.145 min), astragalin (Rt = 26.164 min), lithospermic acid (Rt = 29.590 min), and salvianolic acid (Rt = 30.864 min). In addition, compounds detected at 9.545 min, 28.502 min, and 28.825 min were tentatively identified as benzoic acid and two flavonols according to their UV-Vis spectrum.

As mentioned above, epigallocatechin was the phenolic compound present at the highest concentration in all fractions, with particularly elevated levels in the extract obtained with 75% ethanol (657 mg/100 g extract). This finding is of considerable interest, as the limited prior literature addressing the phenolic characterization of *Borago officinalis* reported the presence of flavanols such as catechin and epicatechin, but not epigallocatechin, in leaf and flower extracts [16]. These results highlight that the phenolic hallmark of the analyzed material depends on multiple factors, including cultivation and processing conditions, the specific plant tissue used (in the present study, leaves and portions of stems that are generally discarded, constituting a mixture that had not previously been characterized in terms of its phenolic profile), the extraction technology applied, etc. [1,35].

Alongside epigallocatechin, rosmarinic acid was the second most abundant phenolic compound in the analyzed samples, reaching its highest concentration in the extract obtained with 50% ethanol (291 mg/100 g extract). In this case, this hydroxycinnamic acid had already been previously detected and identified as a major component in extracts from leaves, flowers, stems, and explants of *Borago officinalis* [16,17]. For both epigallocatechin and rosmarinic acid, UAE applied for 5 min proved to be markedly more effective than conventional extraction at 70 °C for 78 min without ultrasound, yielding significantly higher levels in a much shorter time and at room temperature. This clearly underscores the value of employing this type of advanced and green technology.

Another compound associated with borage is astragalin, a flavonoid that appears to require less polar solvents for efficient extraction. Indeed, it was reported as the most abundant compound extracted with methanol by Michalak et al. (2023) [7], whereas it was recovered to a lesser extent using 25% ethanol in the present study. In contrast, its levels increased in the fractions obtained with 50% and 75% ethanol, reaching values of 85–88 mg/100 g extract.

Based on the results obtained, it is not possible to categorically relate which phenolic species are responsible for conferring higher in vitro antioxidant capacity to the extracts, requiring further studies to reach conclusions. As previously discussed, the correlations between TPC and TEAC values were weak, which suggests the possible involvement of other non-phenolic molecules, such as ascorbic acid, carotenoids, chlorophylls, etc. [37], although the influence of these non-phenolic antioxidants must be specifically validated in these matrices. Moreover, no concentration of any specific phenolic compound showed a strong correlation with the TEAC values calculated for the extracts analyzed by HPLC-DAD. The predominant phenolic compounds in the extracts exhibiting the highest antioxidant capacity -namely, the extract obtained under the optimal RSM conditions (78 min, 70 °C) without ultrasound, and the extract obtained using ultrasound for 45 min- are, in decreasing order of concentration: epigallocatechin, rosmarinic acid, lithospermic acid, and astragalin. All of these compounds are present at significantly higher concentrations (doubling or even tripling the levels) in the extracts prepared with 50% and 70% ethanol, which, however, yielded lower TEAC values in both the DPPH^●^ and ABTS^●+^ assays. Given these difficulties in establishing direct links between individual phenolic compounds and in vitro antioxidant activity, further studies are recommended. On the one hand, a comprehensive characterization of the extracts is needed, not only in terms of their phenolic profile but also regarding their content of other antioxidant molecules. On the other hand, the evaluation of antioxidant activity using cellular and animal models is advisable, as these more accurately reflect physiological conditions in living organisms.

### 3.5. Inhibitory Activity of BPP Extracts Against Bacterial Strains

Using the disc diffusion method, a preliminary screening of the antibacterial potential was performed for the five BBP extracts whose phenolic profiles were analyzed (Table 2 and Table 3). Under the conditions tested, only modest inhibitory effects were detected against *E. coli* and *S. aureus*, while no inhibition was observed for *L. innocua*. Among the extracts, those obtained with 50% ethanol or by UAE for 5 min produced the largest inhibition zones against *S. aureus*, although these differences should be interpreted cautiously given the qualitative nature of the assay. The presence of rosmarinic acid, a phenolic compound that has been extensively studied and is well known for its strong antimicrobial activity [38], and other phenolic compounds may contribute to the observed effects, but no direct association can be established at this stage. These observations are consistent with previous reports describing inhibitory activity of extracts derived from different tissues of *Borago officinalis* (whole plant, leaves, seeds) against *S. aureus*. With regard to activity against *E. coli*, extracts from the whole plant exhibited smaller inhibition zones than those observed against *S. aureus*, whereas no antibacterial effect against this microorganism was detected for leaf- and seed-derived extracts [39,40].

Moreover, the absence of inhibition halos against *L. innocua* in this study, as it has been frequently reported that plant extracts with high TPC values, especially those rich in phenolic acids and flavonoids, tend to exhibit inhibitory activity against *Listeria* species and strains [41]. Indeed, some *Borago officinalis* extracts obtained from flowers or leaves have shown antibacterial activity against *Listeria monocytogenes* strains [20,21]. However, such discrepancies may reflect differences in extract composition, plant material (e.g., inclusion of stems in BBPs), or the specific *L. innocua* strain tested (CECT 8848–SA1 strain). In addition, interactions between phenolic compounds and agar components, as well as the presence of high-molecular-weight and/or less water-soluble phenolic constituents, may restrict their diffusion in the used media and, therefore, their inhibitory action. Furthermore, it is possible that higher extract concentrations are required to reach their minimum inhibitory concentration (MIC). In fact, given that the disc diffusion method provides only an initial qualitative indication of antimicrobial potential, no quantitative or definitive conclusions can be drawn regarding the actual inhibitory capacity of the BBP extracts. Determining MIC, as well as testing additional microbial strains, is therefore recommended for future work to more accurately assess and compare the antimicrobial properties of these extracts.

## 4. Conclusions

Conventional solid–liquid extraction using hydroethanolic mixtures, as well as UAE, proved to be effective technologies for obtaining phenolic-rich fractions from BBPs exhibiting in vitro antioxidant and antimicrobial activities. Although 50% and 75% ethanol were the solvents that yielded the highest TPC values, extractions performed with 25% ethanol produced fractions with the strongest radical-scavenging activity against DPPH^●^ and ABTS^●+^, with the highest TEAC values being recorded for the latter radical. RSM enabled the identification of the most suitable temperature (70 °C) and extraction time (78 min) to maximize extraction yield, TPC, and radical-scavenging capacity. Furthermore, in order to avoid heating and reduce processing time, UAE conducted at 25 °C for 45 min achieved comparable results in terms of TPC and in vitro antioxidant activity. Among the nine phenolic compounds detected in selected BBP extracts, epigallocatechin and rosmarinic acid were the most abundant, particularly in extracts obtained with 50% and 75% ethanol, but also in those extracted with 25% ethanol when ultrasounds were applied for 5 min. Regarding antimicrobial activity, these fractions were unable to inhibit the growth of *L. innocua*, but they produced inhibition halos against *S. aureus* and *E. coli* strains. However, since the disc diffusion method was carried out as an initial approach, further studies must be carried out for a better understanding of antimicrobial actions of BBP fractions, for instance, by measuring MIC or testing alternative microbial strains.

It should be emphasized that all these findings must be interpreted within the context of this being the first study to focus on the use of BBPs based on leaves and portions of stems, obtained in a real food industry setting, as raw material. Extracts were prepared using technologies such as UAE, which remain largely underutilized for species such as borage, and a relevant but preliminary screening of their antioxidant and antimicrobial capacities was conducted. These results encourage further research to continue exploring the bioactive potential of BBP extracts, including a more in-depth assessment of their antioxidant capacity using cellular and animal models that more reliably mimic physiological conditions. In this regard, particular emphasis should be placed on well-designed in vivo studies to validate the biological relevance of the effects observed in vitro, as well as to elucidate mechanisms of action, bioaccessibility, bioavailability, etc. Moreover, while the development of functional foods incorporating these bioactive extracts may represent a promising long-term application, such prospects should be considered with caution. A major limitation of the study of these BBP extracts is the lack of data on PAs concentration, as this quantitative analysis is essential before the application of any borage-derived fractions in foods or nutraceuticals. In addition to sensory evaluation and consumer acceptance studies, comprehensive assessments of stability, safety, effective dosage, and potential interactions within complex food matrices will be required, with in vivo procedures being essential. Altogether, these approaches would provide a more robust scientific basis and could, in the future, support the rational valorization of BBPs within a sustainability and circular economy framework.

## Figures and Tables

**Table 1:** Full factorial 3^2^ experimental design for BBP extraction.

	Independent Factors		Investigated Responses			
Run	Time (min)	Temperature (°C)	Extraction Yield (%)	TPC (%)	DPPH^●^ TEAC Value (µmol/g)	ABTS^●+^ TEAC Value (µmol/g)
1	62.5	47.5	26.8	9.82	35.29	188.47
2	62.5	47.5	26.0	10.29	35.14	185.16
3	120.0	25.0	20.8	10.85	22.59	113.91
4	62.5	25.0	13.2	25.84	41.70	204.33
5	120.0	47.5	22.8	16.00	34.43	170.43
6	5.0	47.5	21.2	14.23	30.11	138.51
7	120.0	70.0	23.6	11.45	37.10	185.52
8	62.5	47.5	25.8	10.54	37.81	192.83
9	5.0	70.0	20.8	12.11	35.83	155.57
10	5.0	25.0	16.8	17.96	45.02	229.60
11	62.5	70.0	22.0	15.27	48.36	241.82

Optimized desirability for all investigated responses: 0.63. Optimal conditions: 70 °C and 78 min. Optimized desirability considering TEAC values: 0.89. Optimal conditions: 70 °C and 78 min. Predicted values at optimal conditions: Extaction yield: 22.7%; TPC: 14.06%; DPPH● TEAC value: 45.44 µmol/g; ABTS●+ TEAC value: 227.60 µmol/g.

**Table 2:** Phenolic composition (mg/100 g extract) of BBP extracts obtained using different extraction conditions (EC). Rt = Retention time. UAE = Ultrasound-assisted extraction.

			mg/100 g Extract				
	Rt (Min)	Compound	1 (EC: 30 Min, 25 °C, Ethanol 50%)	2 (EC: 30 Min, 25 °C, Ethanol 75%)	3 (EC: 78 Min, 70 °C, Ethanol 25%)	4 (EC: 5 Min, 25 °C, Ethanol 25%, UAE)	5 (EC: 45 Min, 25 °C, Ethanol 25%, UAE)
1	9.545	Unidentified benzoic acid ^f^	14.85 ± 0.69 ^ab^	13.79 ± 1.10 ^b^	17.61 ± 0.60 ^a^	16.54 ± 0.31 ^ab^	17.12 ± 0.61 ^a^
2	13.462	Epigallocatechin	559.81± 1.56 ^b^	656.53 ± 7.42 ^a^	223.78 ± 0.96 ^e^	320.67 ± 1.08 ^c^	281.15 ± 3.11 ^d^
3	18.145	Caffeic acid	33.05 ± 0.05 ^b^	36.71 ± 0.21 ^a^	26.22 ± 0.24 ^d^	27.12 ± 0.02 ^c^	19.64 ± 0.08 ^e^
4	26.164	Astragalin	85.49 ± 2.70 ^a^	87.86 ± 0.90 ^a^	36.28 ± 2.13 ^b^	30.89 ± 4.64 ^b^	34.33 ± 2.95 ^b^
5	28.502	Unidentified flavonol (1) ^f^	47.15 ± 20.56 ^a^	48.83 ± 10.55 ^a^	22.03 ± 15.43 ^a^	23.40 ± 20.96 ^a^	23.14 ± 17.01 ^a^
6	28.825	Unidentified flavonol (2) ^f^	41.47 ± 4.23 ^a^	45.25 ± 2.69 ^a^	16.57 ± 0.05 ^b^	9.26 ± 7.10 ^b^	15.52 ± 2.52 ^b^
7	29.204	Rosmarinic acid	290.58 ± 9.50 ^a^	194.11 ± 3.00 ^b^	91.38 ± 4.93 ^d^	114.88 ± 3.62 ^c^	72.37 ± 3.02 ^d^
8	29.590	Lithospermic acid	101.26 ± 6.44 ^a^	67.45 ± 0.50 ^b^	52.57 ± 0.24 ^c^	40.72 ± 2.01 ^cd^	38.80 ± 1.04 ^d^
9	30.864	Salvianolic acid	68.66 ± 4.39 ^a^	47.06 ± 5.72 ^b^	20.42 ± 0.15 ^c^	24.39 ± 2.54 ^c^	26.49 ± 0.17 ^c^
Total			1242.62 ± 24.53 ^a^	1197.58 ± 14.75 ^a^	506.87 ± 16.38 ^c^	607.86 ± 23.16 ^b^	528.58 ± 18.02 ^c^

Rt retention time. ^a–e^ Different letters denote significant statistical differences (*p* < 0.05) for the same compound between different samples. ^f^ A standard was used to corroborate the compound identification and quantification.

**Table 3:** Inhibitory activity of BBP extracts obtained using different conditions against *Escherichia coli* DH5α (EC), *Staphylococcus aureus* CECT86 (SA) and *Listeria innocua* SA1 (LI). Different letters (a–c) indicate statistical significance (*p* ≤ 0.05) between sizes of the inhibition zones (mm) of the same microorganism. “-” = No inhibition zone was observed.

Extraction Conditions				Size of the Inhibition Zones (mm)		
**Time (min)**	Temperature (°C)	Ethanol (%)	Ultrasound-Assisted Extraction (Yes/No)	EC	SA	LI
30	25	50	No	6.43 ± 0.63 ^b^	7.13 ± 0.10 ^bc^	-^b^
30	25	75	No	6.87 ± 0.66 ^b^	6.54 ± 0.26 ^c^	-^b^
78	70	25	No	7.43 ± 0.49 ^b^	6.98 ± 0.37 ^bc^	-^b^
5	25	25	Yes	6.77 ± 0.81 ^b^	7.76 ± 0.29 ^b^	-^b^
45	25	25	Yes	5.99 ± 0.62 ^b^	7.20 ± 0.18 ^bc^	-^b^
Positive antimicrobial control: Gentamicin (10 µg)				14.01 ± 0.89 ^a^	13.88 ± 0.04 ^a^	15.03 ± 0.96 ^a^
Negative antimicrobial control: 25% ethanol (20 µL)				-^c^	-^c^	-^c^

## Data Availability

The original contributions presented in this study are included in the article/Supplementary Material. Further inquiries can be directed to the corresponding author.

## Supplementary Materials

The following supporting information can be downloaded at https://www.mdpi.com/article/10.3390/foods15111917/s1: Table S1: ANOVA table obtained from the Response Surface Methodology analyses considering all responses (extraction yield, total phenolic content, DPPH● TEAC value and ABTS●+ TEAC value), including degrees of freedom (DF) and sources. Same table was obtained considering exclusively TEAC values. Table S2: ANOVA table for the response “extraction yield”, including model statistics and residual diagnostics. DF = Degrees of freedom; A = Time; B = Temperature; PRESS = Predicted Residual Error Sum of Squares. Table S3: ANOVA table for the response “total phenolic content”, including model statistics and residual diagnostics. DF = Degrees of freedom; A = Time; B = Temperature; PRESS = Predicted Residual Error Sum of Squares. Table S4: ANOVA table for the response “DPPH● TEAC value”, including model statistics and residual diagnostics. DF = Degrees of freedom; A = Time; B = Temperature; PRESS = Predicted Residual Error Sum of Squares. Table S5: ANOVA table for the response “ABTS●+ TEAC value”, including model statistics and residual diagnostics. DF = Degrees of freedom; A = Time; B = Temperature; PRESS = Predicted Residual Error Sum of Squares.

## Abbreviations

The following abbreviations are used in this manuscript:

BBP	Borage by-product
UAE	Ultrasound-assisted extraction
TPC	Total phenolic content
TEAC	Trolox equivalent antioxidant capacity
RSM	Response surface methodology
