# Supplementary material for: Extraction of Phenolic-Rich Fractions from Borago officinalis By-Products with Antioxidant and Antimicrobial Activities
Source: Foods. 2026 May 28;15(11):1917. doi: 10.3390/foods15111917 (PMC13256494; doi:10.3390/foods15111917)
Supplement: Supplementary file 1 [file foods-15-01917-s001.zip › foods-4333847-supplementary.pdf]

**Supplementary material**

**Supplementary Table S1.** ANOVA table obtained from the Response Surface Methodology analyses considering all responses (extraction yield, total phenolic content, DPPH$^{\bullet}$ TEAC value and ABTS$^{\bullet+}$ TEAC value), including degrees of freedom (DF) and sources. Same table was obtained considering exclusively TEAC values.

| **Source** | **DF** |
|---|---|
| Model | 5 |
| Residual error | 5 |
| Lack of fit | 3 |
| Pure error | 2 |
| Total (corrected) | 10 |

**Supplementary Table S2.** ANOVA table for the response "extraction yield", including model statistics and residual diagnostics. DF = Degrees of freedom; A = Time; B = Temperature; PRESS = Predicted Residual Error Sum of Squares.

| **Source** | **Sum of squares** | **DF** | **Mean square** | **F-ratio** | ***P*-value** |
|---|---|---|---|---|---|
| A: Time | 11.76 | 1 | 11.76 | 1.36 | 0.2959 |
| B: Temperature | 40.56 | 1 | 40.56 | 4.69 | 0.0825 |
| AA | 0.543439 | 1 | 0.543439 | 0.06 | 0.8119 |
| AB | 0.36 | 1 | 0.36 | 0.04 | 0.8463 |
| BB | 59.9141 | 1 | 59.9141 | 6.93 | 0.0463 |
| Residual error | 43.1979 | 5 | 8.63958 | | |
| Total (corrected) | 164.24 | 10 | | | |

$R^2$ = 73.6983%
Adjusted $R^2$ = 47.3966%
Predicted $R^2$ = 0.0% (PRESS = 329.564)
Standard error of the estimate = 2.93932
Mean absolute error = 1.64498
Durbin-Watson statistic = 2.6304 ($P$ = 0.8886)
Lag-1 residual autocorrelation = -0.428347

**Supplementary Table S3.** ANOVA table for the response "total phenolic content", including model statistics and residual diagnostics. DF = Degrees of freedom; A = Time; B = Temperature; PRESS = Predicted Residual Error Sum of Squares.

| Source | Sum of squares | DF | Mean square | F-ratio | *P*-value |
|---|---|---|---|---|---|
| A: Time | 6.0 | 1 | 6.0 | 0.23 | 0.6493 |
| B: Temperature | 41.7121 | 1 | 41.7121 | 1.62 | 0.2586 |
| AA | 6.5452 | 1 | 6.5452 | 0.25 | 0.6352 |
| AB | 10.4006 | 1 | 10.4006 | 0.40 | 0.5526 |
| BB | 37.2121 | 1 | 37.2123 | 1.45 | 0.2827 |
| Residual error | 128.467 | 5 | 25.6934 | | |
| Total (corrected) | 224.726 | 10 | | | |

$R^2$ = 42.834%
Adjusted $R^2$ = 0.0%
Predicted $R^2$ = 0.0% (PRESS = 985.294)
Standard error of the estimate = 5.06887
Mean absolute error = 2.89612
Durbin-Watson statistic = 2.46532 (*P* = 0.8060)
Lag-1 residual autocorrelation = -0.336924

**Supplementary Table S4.** ANOVA table for the response "DPPH$^\bullet$ TEAC value", including model statistics and residual diagnostics. DF = Degrees of freedom; A = Time; B = Temperature; PRESS = Predicted Residual Error Sum of Squares.

| Source | Sum of squares | DF | Mean square | F-ratio | *P*-value |
|---|---|---|---|---|---|
| A: Time | 47.2643 | 1 | 47.2643 | 2.20 | 0.1978 |
| B: Temperature | 23.9201 | 1 | 23.9201 | 1.12 | 0.3392 |
| AA | 124.582 | 1 | 124.582 | 5.81 | 0.0608 |
| AB | 140.423 | 1 | 140.423 | 6.55 | 0.0507 |
| BB | 83.6817 | 1 | 83.6817 | 3.90 | 0.1052 |
| Residual error | 107.223 | 5 | 21.4446 | | |
| Total (corrected) | 484.412 | 10 | | | |

$R^2$ = 77.8654%
Adjusted $R^2$ = 55.7308%
Predicted $R^2$ = 0.0% (PRESS = 1006.99)
Standard error of the estimate = 4.63083
Mean absolute error = 2.58644
Durbin-Watson statistic = 1.68662 (*P* = 0.2311)
Lag-1 residual autocorrelation = 0.100714

**Supplementary Table S5.** ANOVA table for the response "ABTS$^{\bullet+}$ TEAC value", including model statistics and residual diagnostics. DF = Degrees of freedom; A = Time; B = Temperature; PRESS = Predicted Residual Error Sum of Squares.

| **Source** | **Sum of squares** | **DF** | **Mean square** | **F-ratio** | ***P*-value** |
|---|---|---|---|---|---|
| A: Time | 482.765 | 1 | 482.765 | 0.93 | 0.3786 |
| B: Temperature | 204.984 | 1 | 204.984 | 0.40 | 0.5568 |
| AA | 4815.77 | 1 | 4815.77 | 9.30 | 0.0284 |
| AB | 5302.75 | 1 | 5302.75 | 10.24 | 0.0240 |
| BB | 1583.97 | 1 | 1583.97 | 3.06 | 0.1407 |
| Residual error | 2588.43 | 5 | 517.685 | | |
| Total (corrected) | 13882.8 | 10 | | | |

$R^2$ = 81.3552%
Adjusted $R^2$ = 62.7104%
Predicted $R^2$ = 0.0% (PRESS = 26437.9)
Standard error of the estimate = 22.7527
Mean absolute error = 12.4764
Durbin-Watson statistic = 1.9347 ($P$ = 0.4101)
Lag-1 residual autocorrelation = 0.0145587
